# The Importance of Community-Based and Community-Partnered COVID-19 Testing for Reducing Disparities Among African American Populations

**DOI:** 10.1089/heq.2022.0185

**Published:** 2024-03-07

**Authors:** Chavon Hamilton-Burgess, Jannette Berkley-Patton, Jenifer Allsworth, Carole Bowe Thompson, Frank E. Thompson, Tacia Burgin, Eric D. Williams, Kathryn P. Derose

**Affiliations:** ^1^ Department of Health Promotion and Policy, University of Massachusetts Amherst, Amherst, Massachusetts, USA.; ^2^ Department of Biomedical and Health Informatics, University of Missouri Kansas City (UMKC) School of Medicine, Kansas City, Missouri, USA.; ^3^ Kansas City MO Health Department, Kansas City, Missouri, USA.; ^4^ Department of Psychology, University of Missouri Kansas City (UMKC), Kansas City, Missouri, USA.; ^5^ Calvary Temple Baptist Church, Calvary Community Outreach Network, Kansas City, Missouri, USA.; ^6^ RAND Corporation, Santa Monica, California, USA.

**Keywords:** COVID-19 testing, African American, community networks, health equity

## Abstract

**Background:**

Health inequalities in African American communities have been further exacerbated by COVID-19. Public health departments and other safety-net providers across the United States have partnered with community-based organizations to address barriers to COVID-19 testing in disproportionately impacted communities. This narrative review summarizes lessons learned from published examples of these community-based COVID-19 testing efforts.

**Methods:**

We searched online databases for peer-reviewed articles on community-based COVID-19 testing interventions in the United States aimed at increasing COVID-19 testing among African American populations. We abstracted information about each example and synthesized the primary lessons learned and key aspects that contributed to their success.

**Results:**

Seven examples of community-based COVID-19 testing aimed at increasing testing among African Americans and other underserved populations were identified and described, across various U.S. locations and involving multiple types of partners (1) St. Paul, MN (faith, health centers, Mayo Clinic); (2) Chicago, IL (university hospital and health centers); (3) NC (health centers, Community Advisory Board); (4) Baltimore, MD (hospitals, community clinic, mobile clinic); (5) Marion County, FL (health department and community partners); (6) New Orleans, LA (health department and health system); and (7) New York City, NY health and hospital system, mobile clinic).

**Discussion:**

Several key aspects of the COVID-19 testing models included the following: (1) close proximity of the testing site to affected communities and availability of walk-up and drive-through testing options; (2) partnerships between safety-net providers and broad community networks, which facilitated outreach and trust; (3) increased resources for safety-net providers; and (4) the use of data to identify areas of need and track impact. The merging of resources and relationships among well-equipped, safety-net providers and other health care institutions and culture-rich, community-centered organizations, to jointly address structural and systemic inequities, is key to cultivating health equity in the distribution of COVID-19 testing and other essential public health services.

## Introduction

The United States has had the most cases and deaths from COVID-19 in the world, 103,804,263 and 1,123,836, respectively, as of March 10, 2023.^1^ Although COVID-19 has affected all racial groups across diverse locations and stages of the pandemic, the virus has disproportionately affected racialized and minoritized populations, with African American communities experiencing the greatest impact.^2^ For example, even early in the pandemic (June 2020), data showed that in Maryland, African Americans made up 31% of the population but accounted for 52% of the deaths from COVID-19; in Michigan, African Americans were 14% of the population but 40% of the COVID-19 deaths; in Louisiana, African Americans accounted for 70.5% of deaths while comprising 32% of the population.^3^

These inequalities, although disturbing, are tragically not surprising, as health disparities in African American communities existed long before the COVID-19 pandemic. African American populations experience disproportionate rates of chronic illness, which exacerbated their susceptibility to COVID-19. Health conditions that increase COVID-19 risk, such as hypertension, peripheral arterial disease, and type II diabetes, have been found to be twice as high in African American populations compared with white populations, even when controlling for risk behaviors (e.g., tobacco use, alcohol consumption).^2^ In addition, African American communities face ongoing environmental and employment risks that place them at greater risk to contracting COVID-19.

To really understand the causes of these health inequalities, one must consider the structural racism that African American communities experience. As noted by Egede and Walker, structural racism, or “the ways in which societies foster discrimination through mutually reinforcing inequitable systems,”^4^ affects health through multiple paths, including social deprivation, increased environmental exposures, inadequate access to quality health care, and injury and trauma from state-sanctioned violence.^5^ An example of structural inequities are the persistent disparities in health insurance coverage by race/ethnicity. The federal government has acknowledged that a major barrier to health care access is inadequate health insurance coverage.^6^ To compound this issue, even when health insurance is obtained, African Americans may face implicit and explicit racial biases from providers, which may result in poor quality care and medical mistrust, affecting health care screening, adherence to treatment, and future care-seeking.^7–9^

Medical mistrust among African American populations originates from years of mistreatment and racist interactions within the health care system and continues today evidenced by lack of acknowledgment of medical concerns or poor communication during medical visits.^8^ Although often framed in the literature as an issue to be addressed within the African American community, the solution to medical mistrust lies in addressing the systemic inequities experienced by this group of people.^10^

In terms of the social deprivation path to COVID-19 inequalities, African Americans are overrepresented among service industry workers, who, during COVID-19, were deemed essential workers and thus experienced disproportionately higher levels of COVID-19 exposure because of high levels of public interaction, their inability to work from home, and greater likelihood of using public transportation. Furthermore, workers in the service industry face more barriers to COVID-19 testing and care, given that they often do not have paid sick time and experience gaps in health insurance, reflecting inadequate access to health care.

Adequate access to and utilization of COVID-19 testing, especially for highly impacted groups such as African American populations, are essential for pandemic control, as they facilitate early detection, self-isolation, and prevention of onward transmission.^11^ Particularly during the early stages of the pandemic, COVID-19 testing was woefully inadequate, particularly for communities of color, due to barriers limiting access to testing sites and services. For example, North St. Louis, a predominately black community that was one of the areas with the highest infection rates in Missouri, did not have a single testing site several weeks into the pandemic; perhaps not surprising, then, that data from March to September 2020 found that black populations had consistently lower COVID-19 testing rates per diagnosed cases compared with white populations in two Missouri regions.^11^

In New York City, African American and Hispanic communities experienced higher rates of disease and deaths compared with whites, but even after dramatically increasing the testing sites in black and Latino areas, in May 2020, more testing sites (39%) were located in predominately white zip codes compared with black (17%) and Latino (17%) areas.^12^ Participants involved in focus groups conducted in urban and rural Alabama expressed concerns regarding the lack of access to reliable transportation, perceived cost of the test, and medical mistrust as deterrents to obtaining a test outside of their community.^13^

Now 3 years since the advent of the pandemic and even with widespread availability of COVID-19 vaccinations, COVID-19 testing remains an important tool in controlling the pandemic. Identifying best practices on how to provide community-based, accessible COVID-19 testing in ways that are acceptable to the most vulnerable populations is critical. Community-based testing models have been successful in addressing barriers to testing for other types of health issues that disproportionately affect African American communities, such as HIV and other sexually transmitted infections.^14,15^ For example, studies have found that community-partnered HIV testing interventions with African American faith communities in Kansas City and Los Angeles—offering church-based HIV testing and a supportive environment for testing—can improve testing dramatically.^16,17^

Thus, we examined the current literature for examples of community-based COVID-19 testing initiatives to identify lessons learned that might inform broader scale implementation of community-based testing strategies.

## Methods

Articles were identified using various combinations of the following search terms in PubMed and Academic Search Premier: interventions, COVID-19, testing, African Americans/blacks, health disparities, health equity, community-based, and access. The searches identified 100 articles; abstracts were reviewed for relevancy to the subject matter (community-based COVID-19 testing to reduce disparities among African American populations in the United States), identifying 17 articles. Abstracts were excluded if the research and/or information pertained to locations outside of the United States or did not discuss community-based testing for COVID-19. After full-article review of the 17 articles, 7 articles were selected that described community-based COVID-19 testing interventions with African American populations (the other 10 articles discussed the topic but were not interventions).

## Results

In this study, we summarize the seven examples of community-based COVID-19 testing with African American populations: (1) the FAITH!-OCHC partnership in St. Paul, Minnesota; (2) the University of Illinois Hospital (UIH)/Miles Square Health Center (MSHC) project in Chicago, Illinois, (3) the North Carolina Health System Initiative in North Carolina; (4) Baltimore Mobile Health Clinic Pilot; (5) Marion County Health Department COVID-19 Testing Intervention; (6) New Orleans Health Department (NOHD) and Louisiana Children's Medical Center (LCMC) Walk-Up Testing Project; and (7) NYC Test and Trace mobile testing project. Each example includes the geographic location(s), the population(s) served, partners involved (if applicable), the model that was used, and outcome data (if reported in the article). The examples are summarized in Table 1 and described briefly below.

**Table 1.** Community-based COVID-19 testing examples

Project name	Geography and population(s)	Length of intervention	Intervention description	Outcomes
FAITH!-OCHC^18^	Minneapolis-St. Paul; racial/ethnic minorities and low-income frontline workers	10 weeks	Drive through at a community health clinic organized by a multidisciplinary team of medical professionals	2006 tests were performed. At week 4, the positivity rate was 46% and at week 10 it was 8%.
University of Illinois Hospital/Miles Square Health Center^19^	Chicago; primarily in African American communities	5 months	Drive -hrough and walk-up testing at a community health clinic	7523 tests were performed, 5289 for drive-through and 2234 for walk-up. Walk-up sites showed a slightly higher positivity rate, an overall 19.3% of tests being COVID positive, compared with an 18.1% positivity rate for the drive-through site.
North Carolina Health System Initiative^20^	North Carolina (13 counties); underserved communities of color (79.2% identifying as nonwhite)	None available	Mobile testing in churches, community centers, food processing plants, homeless services agencies, shopping centers, farms	64,442 people tested; positivity rate 18%, which was higher than state positivity rate.
Life Bridge Mobile Clinic Pilot Project^22^	Baltimore, MD (west Baltimore); hard to reach and marginalized individuals at highest risk for adverse outcomes due to COVID-19 (84% of those tested were black)	6 weeks	Mobile Clinic visits that included medical assessments and COVID-19 PCR testing	288 PCR tests were performed.
Marion County Health Department COVID-19 Testing Intervention^21^	Indianapolis, Indiana; populations disproportionately impacted by the COVID-19 pandemic	9 months	Drive-through and walk-up testing at churches and other community sites identified through data and community partners	Rates of new cases declined for focal racial groups after targeted testing sites and information campaigns began (black, Latino, and Burmese residents).
New Orleans Health Department and LCMC Walk-Up Testing Project^23^	New Orleans Parish; low-income minority communities	2 months	Walk-up testing with the New Orleans City lines	The percentage of people tested who were residents of Orleans Parish increased from 46% to 78%. The percentage of the population tested within a census tract increased from 1% to 2%.
NYC Test and Trace mobile testing project^24^	New York City; neighborhoods with highest COVID-19 positivity rates (blacks 31% and Hispanics 48% of those tested)	5 months	Outdoor testing, vehicle testing, and expanded vehicle testing	This intervention provided testing to 150,351 unique patients and processed 274,083 tests in total.

FAITH, Fostering African American Improvement in Total Health; LCMC, Louisiana Children's Medical Center; OCHC, Open Cities Health Center; PCR, polymerase chain reaction.

### FAITH!-OCHC

This partnership involved Fostering African American Improvement in Total Health (FAITH), a community-based participatory research program on health and wellness within African American communities, Open Cities Health Center (OCHC), a federally qualified health center (FQHC) serving socioeconomically disadvantaged populations, and the Mayo Clinic in Minnesota.^18^ OCHC provides care for more than 10,000 patients annually. Their patient population is racially and ethnically diverse, predominantly low-income, and underinsured or uninsured (81%). A large proportion are African American (47%), of low English proficiency (17%), and immigrants or refugees. A majority are essential workers in public and service sector occupations (long-term care facility workers, grocery store clerks, delivery drivers) and reside in overcrowded neighborhoods with multigenerational households.^18^

The FAITH!-OCHC partnership was established due to concerns about disproportionately high rates of COVID-19 cases among African Americans in Minnesota and lack of accessible testing. The project aimed to provide easily accessible COVID-19 testing to medically underserved populations and to mitigate community spread. The team disseminated information about the community-based testing site through OCHC's website as well as culturally sensitive texts and mailings to health center patients. A dedicated triage line was established for patients experiencing COVID-19 symptoms and the community-based testing site was near a public transportation route.^18^ Patients received their results within 24–48 h, which was supplemented with appropriate guidance if they were positive, to isolate to minimize further transmission. Beginning in May of 2020, they established a drive-through COVID-19 testing lane at the OCHC medical facility, which provided culturally sensitive primary care to residents of seven counties in the Minneapolis–St Paul metropolitan area.

At the conclusion of the 10-week project period, a total of 2006 tests had been completed, or ∼200 per week. Early on during the project, the positivity rate peaked at 46%, but by week 10, it had declined to 8%.

### UIH/MSHC

This project was a collaboration between the UIH in Chicago and MSHC, a network of FQHCs run by UIH. MSHC has been providing care to neighborhoods in Chicago for more than 50 years and has 14 practice sites, including 6 community health clinics, 7 school-based health centers, and 1 clinic in Rockford.^19^

The aim of the UIH/MSHC COVID-19 testing project was to increase testing capacity in the most affected racial/ethnic minority communities, which were identified as the West and South sides of Chicago, which are primarily black communities.^19^ In March of 2020, UIH and MSHC set up an outpatient testing response, inclusive of drive-through as well as walk-up testing capability. The team used the Plan-Do-Study-Act model, which allowed for quickly planning, implementing, evaluating work done, and making changes real time.

Guided by this model, subsequent site modifications and the establishment of new sites occurred. For example, two additional drive-through sites, in the Near West side and Southwest side, opened by April 2020, and six walk-up testing sites were incorporated to provide access to those without cars.^19^ The walk-up sites were based in FQHCs in highly segregated racial/ethnic minority communities, A total of 63.5% of those tested at the walk-up sites were black, compared with 43.9% of individuals tested by the city of Chicago.^19^

Over 5 months, this collaboration completed 7523 tests, 5289 at drive-through sites and 2234 at walk-up sites. Overall, walk-up sites had a 19% COVID positivity rate in comparison with the 18% positivity rate at drive-through sites. The positivity among age groups was consistent with Chicago rates, see the decreasing rates with age.^19^

### North Carolina Health System Initiative

This initiative included six health centers, Novant Health, Atrium Health, Neighborhealth, Cone Health, UNC Rex Wake County, and UNC School of Medicine. The initiative deployed mobile testing across 13 counties in North Carolina. Each mobile site was chosen through input from community partners and/or state and GIS data. For example, UNC Rex health developed a Community Advisory Board composed of organizations that served black and Latino individuals, to help identify testing sites in Southeast Raleigh. Neighborhealth partnered with community-based organizations and used state data to identify high poverty areas.^20^ After identifying sites, each health system leveraged community partnerships to host testing in trusted community spaces such as churches, community centers, food processing plants, homeless service agencies, shopping centers, and farms.

With the help of community stakeholders, UNC health systems developed messaging to inform communities about the testing sites. Information about the sites was disseminated through social media, community networks (word of mouth), support from recording artists, a Spanish language helpline, and online tools. In addition, Atrium Health developed a taskforce to develop culturally appropriate communication to engage minority communities; this task force was independent of the Community Advisory Board established by UNC Rex Health.

All COVID-19 testing sites included in the North Carolina Initiative offered wraparound services such as interpreters to facilitate whole-person care and screenings for social drivers of health such as food security, housing, and behavioral health. Care coordination to community services and the establishment of medical homes were provided by several of the sites at either their institution or a partner clinic. All programs worked closely with local health departments to ensure that those who tested positive were contacted for case investigation and contact tracing.

A total of 64,442 individuals (79% nonwhite) were tested across all sites, with an 18% positivity rate. Because this rate was higher than the rates for the state of North Carolina, it illustrated the success of the project being able to reach the residents at highest risk.^20^

### Baltimore Mobile Health Clinic Pilot

This pilot project took place on the west side of Baltimore, Maryland, and was led by Life Bridge Health, a major health provider that manages hospitals throughout Greater Baltimore. This organization sought to pilot a Community Mobile Health Clinic program to provide health services, including COVID-19 testing, to hard-to-reach populations, which included black residents.^21^ They used a vulnerability index developed by the Maryland Department of Public Health and Socially Determined, Inc., to identify highest risk community members by location, who were then contacted for mobile clinic appointments. The mobile clinic included two vans with drivers, a registered nurse, and a community health worker supported by clinicians at Life Bridge's “virtual hospital,” a provider-staffed triage and care support arm of the health system.^21^

The pilot program confirmed 343 primarily Medicare patients to participate in the mobile clinic. Three hundred sixteen patients were assessed and 288 consented to COVID-19 polymerase chain reaction (PCR) tests during the 6-week period (June to July 2020); 8% of patients canceled their appointment. Of the patients served at the mobile clinic, 84% identified as black.^21^

In addition, the program measured patient satisfaction as well as the proportion of patients with chronic health conditions to understand the usefulness of this model postpandemic. Ninety-three percent of patients reported being very satisfied or satisfied with services at the mobile clinic. Many patients reported being diagnosed with various chronic health conditions such as hypertension, diabetes, and asthma, among others.

### Marion County Health Department COVID-19 testing intervention

This intervention began in April 2020, led by the Marion County Health Department (FL). COVID-19 surveillance data were utilized to identify community testing sites in high-need areas, defined as areas disproportionately affected by the COVID-19 infection.^22^ The health department matched case reports from public and private laboratories to electronic health records to create a community dashboard. The sites established were in Indianapolis, Indiana, some temporary and others more long-term, according to COVID-19 infection data. Once a neighborhood was established as a hotspot, the health department partnered with community-based organizations, churches, and community clinics to identify testing sites within that area.^22^ The health department continued these partnerships throughout the duration of the intervention.

Testing sites included in-person registration, to ensure that people who lacked access to the internet were able to access services. The testing sites were walk-up and drive-through and near public transportation. In addition, testing services were available on the weekend to account for varying work schedules. In response to this intervention, the case and testing disparity gap between the most vulnerable populations, black, Latino, and Burmese, decreased in comparison with white populations in Indianapolis.^22^ Although the community-based testing intervention will not be sustained postpandemic, the health department will continue using surveillance data to identify and address health disparities experienced by vulnerable communities in Marion County.^22^

### NOHD and LCMC Walk-Up Testing Project

This project took place between May and June 2020. The NOHD partnered with LCMC, a local health care network, to improve COVID-19 testing services for low-income and minority communities by hosting walk-up sites throughout the city of New Orleans. Mobile testing sites were selected through the identification of communities that were at highest risk of contracting COVID-19 and were advertised through the media and at the neighborhood level. Spanish and Vietnamese language interpreters were available at most sites.^23^ Mobile sites operated on a rotational basis, remaining in neighborhoods for 2 to 3 days before moving on to the next neighborhood. NOHD and LCMC also used geocoding (patients' home address, race, age, and gender) to understand the distance that patients traveled to obtain a COVID-19 test and the nearest testing site they could potentially have gotten tested.^23^

Over 2 months, 9721 tests were performed. The transition from drive-through testing to walk-up sites increased the percentage of Orleans Parish residents who got tested from 46% to 78%. The mean percentage of the population in each census tract getting tested doubled from 1% to 2%. Overall, only 20% visited the closest testing site to their address. Patients on average traveled an additional 3.1 miles to get tested.^23^ African American and Asian patients were more likely to travel the least distance to get tested compared with white patients. Hispanic residents were more likely to travel further distances to obtain a test, partially due to living in the suburbs of New Orleans.

### NYC Test and Trace Mobile Testing Project

This project took place between December 1, 2020, and April 1, 2021, with the aim to reduce COVID-19 testing inequities. Test and Trace was established by NYC Health and Hospitals guided by the following three pillars: COVID-19 testing, contact tracing, and “take care” (isolation and after care support).^24^ The Test and Trace team sought to address testing inequities among black/African American and Hispanic communities. They developed mobile testing sites in collaboration with community partners, which included houses of worship, small retailers, government and contracted service providers, and traditional community-based organizations.^24^ Partners were identified through an online survey, the Test and Trace advisory board, partners in the NYC Care program, and traditional governmental referral mechanisms. Test and Trace managed the operations of the mobile sites while community partners led outreach, testing site placement, and direct selection of testing locations.

The project consisted of clinician-administered and patient-administered sites. The clinician-administered sites had the following three phases: (1) outdoor testing (June to November 2020), 14 clinical teams offered free PCR testing, M-F, at locations under pop-up tents; (2) vehicle testing (November 2020 to April 2021), 8 teams and an enclosed testing vehicle; and (3) expanded vehicle testing (December 2020). Test and Trace engaged an additional vendor to provide both vehicle and mobile testing services and was scaled to 40 mobile units by mid-March 2021. Patient-administered testing took place in community-based spaces such as churches and senior centers. This consisted of staff trained in self-swab testing guiding patients in collecting their sample using home test kits, packaging the sample, and sending the samples to the laboratory. Patients were contacted by the laboratory through the email that they provided during registration.^24^

Testing locations were selected according to elevated positivity rates, community partner recommendations, and existing city resources such as parks and public housing campuses.^24^

Over 5 months, the Test and Trace mobile testing project served 150,351 unique patients and processed 274,083 tests in total, of which 6% returned a positive result. The model accomplished its goal of improving testing access to communities. For example, self-identified black/African American residents comprise 24% of NYC's population but 31% of completed clinician-administered tests. Similar statistics for self-identified Hispanic residents were evident in the data (29% of the total NYC population, but 48% of the completed clinician-administered tests).^24^

## Discussion

Across the United States, public health departments and other health providers have implemented diverse approaches to increase accessibility to COVID-19 testing. In this study, we have identified and explored seven examples of community-based COVID-19 testing from the literature that specifically aimed to increase the reach of COVID-19 testing among African American or black populations. Review of these models has yielded several key issues or aspects that potentially contributed to their success: (1) close proximity of the testing site to the affected communities and the availability of both walk-up and drive-through testing options; (2) partnerships between safety-net providers and broad community networks, which can facilitate outreach and trust to increase access to and use of COVID-19 testing; (3) increased resources for safety-net providers; and (4) the importance of data to identify areas of need and track impact. We expand on these lessons below to inform community-based COVID-19 testing strategies going forward.

### Testing accessibility

Regarding testing locations, the interventions were placed in communities that were impacted disproportionately by COVID-19, and particularly in community settings that are frequently accessed (e.g., churches, shopping areas) and, in the case of mobile vans, in the residential areas most affected. This strategy helped address transportation issues, which are a barrier to accessing health services for underserved populations, especially African American communities.^13^ In addition, walk-up testing was offered to increase accessibility for those who may not have private vehicles. Walk-up testing and on-site registration can also greatly facilitate access for persons who may not be able to preregister for testing services due to limited access to data-driven devices such as computers or smartphones, broadband internet, as well as limited skills to effectively manage COVID-19 online registration platforms.^20^

### Community-based partnerships

In terms of partnership and facilitating trust, the UIH, FAITH!, NYC Test and Trace project, and New Orleans Department of Health projects implemented testing through trusted community entities, MSHC, OCHC, and LCMC, respectively. These safety-net providers have long tenures serving their racially and ethnically diverse, predominantly low-income, and underinsured or uninsured communities. They have extensive networks of care within underserved communities through established community clinics and school-based health centers.

Nevertheless, opening testing sites in under-resourced communities in and of itself may not be enough to increase testing participation. All seven COVID-19 testing efforts described the above-involved partnerships between safety-net providers and/or public health departments and broad community networks such as faith-based organizations. Introducing testing in partnership with trusted institutions can enable the care to be more culturally relevant, increases the trustworthiness of public health and health care providers among the African American population, and facilitates uptake, follow-up, and sustainability.^25–27^

### Adequate resources

Although financing of community-based COVID-19 testing was not addressed explicitly in these published examples, federal policies and funding undoubtedly have played an important role. Increased financial support for testing and programmatic funding, for example, through the Department of Health and Human Services–Center for Medicare and Medicaid, one of the primary sources of funding for community health centers, is necessary to enhance the capacity of the safety-net providers and hospitals that play a valuable role in reducing health inequities. As cited by Yearby et al., such organizations are under-resourced and financially constrained and score lower on patient satisfaction surveys, underperform on evidence-based metrics, and report higher rates of adverse safety events and complications, which perpetuate health inequities and inequalities in African American communities.^6^

Furthermore, studies have found that African American or black communities with high levels of poverty are often less likely to be designated medically underserved areas, even though they meet these definitions, and thus are less likely to have FQHCs.^28–30^ The result of the lack of sustainable funding is currently illustrated nationally, due to the dwindling of federal COVID-19 relief funds used to reimburse community health centers and hospitals for interventions that minimized the spread of COVID-19. In addition, funding through research mechanisms such as the National Institutes of Health (NIH), National Health Foundations, or academic funding is critical to better understand disparities and promising models for addressing them.

### Data accessibility

Finally, accessibility of COVID-19 data was an essential step in the development of all the COVID-19 testing interventions to effectively identify areas of greatest need for COVID-19 testing and impact. The Marion County Health Department and the NYC Test and Trace used local positivity rates in conjunction with other resources such as community partner recommendations, to decide where to place testing sites; Marion County also used race-specific testing rates over time to evaluate their strategies.^24^ A lack of information about COVID-19 disparities at different points during the pandemic and regarding testing hampered strategies to ensure equitable access to COVID-19 testing. For example, during August 2021, all 50 states reported COVID-19 infection and death rates by race/ethnicity, whereas only 8 states reported COVID-19 testing rates by race/ethnicity, which directly impacts the ability to allocate COVID-19 testing resources where needed.^31^

The COVID-19 Health Equity Dashboard, a tool developed by Emory University, shows the number of cases and deaths for each U.S. county by age, race, ethnicity, employment status, poverty, and length of commute to the nearest hospital.^32^ This kind of stratified information helps to identify areas with the greatest COVID-19 burden and enables resources to be directed toward these communities.^5^ Having similar information for COVID-19 testing would help direct strategies and resources to subgroups within communities who are most affected and vulnerable.

The interventions described in the examples illustrated success as they were able to address COVID-19 testing access barriers, including hesitancy and proximity to the community, but there were important omissions in their descriptions that may impede replication. The articles provided limited detail about the follow-up process, including information about the staff who provided the test results, how a positive result was managed, and the communication between testing staff and the patient's primary care provider (if applicable). Examples with more detailed information could enable elected officials and public health administrators to advocate for financial resources and/or the development of policy and replicate successful models with adequate capacity.

Nevertheless, the examples reviewed provide important lessons about successfully expanding access to COVID-19 testing for African Americans and other high-risk and underserved populations, which can be useful for ongoing efforts to provide testing and vaccinations for COVID-19 and other critical health issues. In the midst of a public health emergency, real-time data on testing and outcomes are essential for identifying and prioritizing communities most in need, tracking changes over time (particularly in response to interventions), and identifying new strategies to increase access to and uptake of testing. Having ongoing community-level data that are broken down by racial/ethnic groups can also serve as a measure of accountability for health care systems' commitments to achieve health equity.^11^ Proximity of testing and availability of both walk-up and drive-through options are also key to consider.

Finally, the merging of resources and relationships among well-equipped safety-net providers and other health care institutions and culture-rich, community-centered organizations, to jointly address structural and systemic inequities in underserved populations, such as African American communities, is key to cultivating health equity in the distribution of COVID-19 testing and other public health services.^18^ Strong partnerships with trusted community organizations are essential in building systems that result in health equity for all communities.^24^

## Abbreviations Used

FAITH: Fostering African American Improvement in Total Health
FQHC: federally qualified health center
LCMC: Louisiana Children's Medical Center
MSHC: Miles Square Health Center
NOHD: New Orleans Health Department
OCHC: Open Cities Health Center
PCR: polymerase chain reaction
UIH: University of Illinois Hospital
